# Strength properties and ability to dissipate mechanical energy of biopolypropylene basalt/cellulose composites with the addition of antibacterial turmeric

**DOI:** 10.1038/s41598-023-51145-6

**Published:** 2024-01-08

**Authors:** Karina Rusin-Żurek, Stanisław Kuciel

**Affiliations:** Faculty of Materials Engineering and Physics, Cracow University of Technology, Kraków, Poland

**Keywords:** Engineering, Materials science

## Abstract

The aim of this study was to evaluate the possibility of producing novel reinforced biocomposites based on polypropylene produced from biomass with the addition of antibacterial turmeric as a natural antibacterial agent for the manufacturing of orthoses and other small external medical equipment. Six hybrid composites containing 5–15% basalt fibers, 5–15% microcellulose fibers, 2% turmeric powder and 2% anhydride maleic compatibilizer were produced on a biobased polypropylene matrix by injection molding. The basic strength properties were determined in a static tensile, bending and impact test. The low-cycle dynamic test was carried out to determine changes in dissipation energy and the development of relaxation processes. In order to assess the microstructure of the composites, SEM micrographs were taken after the tensile test. The obtained results confirm that it was possible to produce functional biocomposites based on biopolypropylene with the addition of basalt and lignocellulosic fibers modified with natural antibacterial turmeric. Based on the results of strength properties tests, it can be seen that the addition of basalt fibers increases strength and stiffness, while microcellulose particles reduce the ability to dissipate mechanical energy, and in both cases water has a plasticizing effect on the produced composites. The addition of fibers increases the flexural modulus by 39–196% and is higher the higher the fiber content. The most promising seem to be hybrid composites with a balanced proportion of 10:10 and 15:15 basalt and EFC fibers, which are characterized by 20% higher strength and almost two and a half times higher stiffness than neat polypropylene.

## Introduction

In order to protect our planet, it is necessary to use modern materials that do not to cause natural environment risk. For many years, the area of interest of scientists has been the development of innovative composites that could replace traditional materials^1,2^. Materials such as PP, PET or PE made from bio-based raw materials have similar properties as those conventional made from petroleum^3^. Efforts are being made to improve the properties of bio-based materials, such as durability, heat resistance and flexibility^4,5^. Consumer awareness has led to greater attention being paid to the production of green materials with a reduced carbon footprint that are biobased and biodegradable^6,7^. In 2022, the global production of bioplastics consisted of 51.5% biodegradable and 48.5% biobased/non-biodegradable plastics, including 3.9% PP. It is expected that by 2027 the production of polypropylene will increase to 6%^8^. Polypropylene is a linear hydrocarbon polymer, its properties include low density, high stiffness, heat resistance, chemical inertness, steam barrier properties (food protection), good transparency, elasticity and recyclability^9–12^. All this make PP the second most important polymer after PE used in fields such as packaging production, chemical and pharmaceutical industries, construction, automotive but also in medical engineering, wind turbines and pressure vessels application, which will further stimulate the demand for polypropylene products^13–16^.

To obtain biocomposites with increased strength and stiffness properties, the addition of biodegradable natural or mineral fibers seems to be an ideal solution^17,18^. Composites with such fillers are very popular due to low weight, high strength, durability and corrosion resistance^17,19^. Examples of such fibers are cellulose, flax, hemp, kenaf, jute, agave but also mineral fibers like basalt or mica.^20–23^. Gomez-Caturla et al. developed biobase polypropylene/mango peel flour (bioPP/MPF) composites using extrusion and injection molding processes^24^. Compatibility between bioPP and MPF was improved by using PP-g-IA as compatibilizer and dicumyl peroxide. The results showed that composites with MPF (30 wt%) and the addition of a compatibilizer achieved a higher Young’s modulus. The elongation at break also showed very promising results with a maximum value of almost 30%. These additives also improved thermal stability and crystallinity. The mechanical results were verified on SEM images, where a very narrow gap was observed between the MPF particles and the bioPP matrix. To study the effect of lignocellulosic fibers on deformation and failure of PP composites Renner et al. prepared PP/wood composites from two lignocellulosic fibers (Filtracel EFC 1000, d = 210 μm and Arbocel CW 630 PU, d = 30 μm) with different particle sizes^25^. The wood content was varied from 0 to 80 wt%. Maleinated polypropylene (MAPP) was added to improve interfacial adhesion. Results have shown that the stiffness of the composites increased significantly with the increase in the content of lignocellulosic fibers. Neat PP reached a tensile strength value of 17.5 MPa, while PP with the addition of 10% EFC 25 MPa (increase by 47%), with 20% EFC 30 MPa (increase by 77%), and with 40% almost 40 MPa (135%). For PP with the addition of CW fibers and compatibilizer, these values increased by similar percentages, but these composites reached slightly lower values. It was very similar in the case of Young’s modulus, where the increase of this parameter depending on the fiber content, as in the case of tensile strength, was almost linear. At larger filler content, a few points deviate from the general tendency indicated by the solid line. Wang et al. investigated and compared the mechanical properties and thermal stability of polypropylene composites reinforced with basalt fibers (BFRPP) and polypropylene^26^. Compared with PP, the tensile strength and Young’s modulus of PB10 (PP with 10% addition of basalt fibers) improved by 57% and 225% respectively. The tensile strength and Young’s modulus of PB20 (PP with 20% addition of basalt fibers) have been improved by 91% and 315%, respectively. However, the elongation of PB10 and PB20 was significantly reduced. Results showed that the addition of basalt fibers to the PP matrix can improve the mechanical properties of PP, but also increase the brittleness of the matrix.

Currently, there are only a few studies on the mechanical properties of hybrid polypropylene composites with the addition of basalt and cellulose fibers. One of them is work^27^ where Song et al. studied (BF)-reinforced polypropylene (PP) composites based on the synergistic reinforcement of cellulose nanocrystals (CNCs). The fibers were modified with a silane coupling agent, thanks to which higher reinforcement effects were achieved. The study shows that when the mass percentage of CNCs and BFs are 1 and 30%, respectively, the composite achieves the highest mechanical strength, which is 64.31% higher than of the neat PP. CNCs not only promoted the improvement of PP crystallinity by heterogeneous nucleation but also formed a wedge shaped structure between them and BFs through hydrogen bonding to prevent PP molecular movement. In another study the effects of two different coupling agents, endowed with maleic anhydride (MA-g(grafted)-PP) and acrylic acid (AA-g-PP) functionalities, on the composite properties were investigated as a function of their amount^28^. For hybrid composites with basalt/cellulose (15/15) and 5 wt% MA-g-PP, significant increases in tensile strength and modulus, flexural strength and modulus, and impact strength were achieved by 45% and 284%, 97% and 263%, and 13% compared to neat PP, respectively.

Due to the epidemiological threat and consumer interest, the great advantage of materials is their additional antibacterial properties Such composites can be obtained by adding antibacterial particles to the matrix by injection molding, blow molding or extrusion techniques^29^. Examples of such fillers are metal nanoparticles of silver, gold, copper, zinc but also natural substances, e.g. horseradish, garlic, turmeric or oregano, whose antibacterial properties have been confirmed in many studies^30–33^. Turmeric (*Curcuma* *longa*) rhizome has been traditionally used as antimicrobial agent as well as an insect repellant^34,35^. Several studies have reported the broad-spectrum antimicrobial activity for curcumin including antibacterial, antiviral, antifungal, and antimalarial activities^36^. The antibacterial properties of curcumin^37^ have been tested against 100 strains of pathogens belonging to 19 species. Tests showed greater sensitivity Gram-positive than Gram-negative bacteria. Curcumin has proven to be effective against certain bacteria and strains (Streptococcus pyogenes, S. aureus, Acinetobacter lwoffii, Enterococcus faecalis and Pseudomonas aeruginosa). On other strains also showed antibacterial effects, but in a very high dose. In addition to studying the antibacterial properties of turmeric, Sunthar et al. also studied its effect on mechanical properties^38^. Composites based on polyethylene with the addition of turmeric were produced. Microscopic examinations showed that the maximum the amount that can be added to the composite is 5 wt%. In case of adding more improper dispersion occurred, resulting in an inhomogeneous and brittle composite. In vitro tests were carried out on E. coli and S. aureus bacteria and Candida albicans fungus. Studies have shown antibacterial activity against E. Coli and S. aureus. The tensile test showed that the addition of turmeric slightly reduced the mechanical properties of polyethylene. The antifungal activity and mechanisms in vitro of natural essential oil (EO) derived from turmeric (*Curcuma longa* L.) against Aspergillus flavus have also been investigated^39^. The antifungal activities of turmeric EO on the mycelial growth, spore germination and aflatoxin production were observed. The antifungal effect was associated with disruption of the inner membrane system of fungal cells and mitochondria.

The aim of work was to produce novel composites reinforced with natural fibers based on biopolypropylene with antibacterial properties. According to available sources, no research on such composite has been carried out yet.

Our concept is the production of green composites, which can be bio-based, naturally colored and naturally antibacterial. The new antibacterial biopolypropylene matrix composites modified with lignocellulosic particles and reinforced with natural basalt fibers can be used for the injection molding of medical device components such as small orthoses and external bone deposition plates. These products will feature unique antibacterial properties based on curcumin and increased strength and stiffness thanks to basalt fibers.

**Figure 1:**
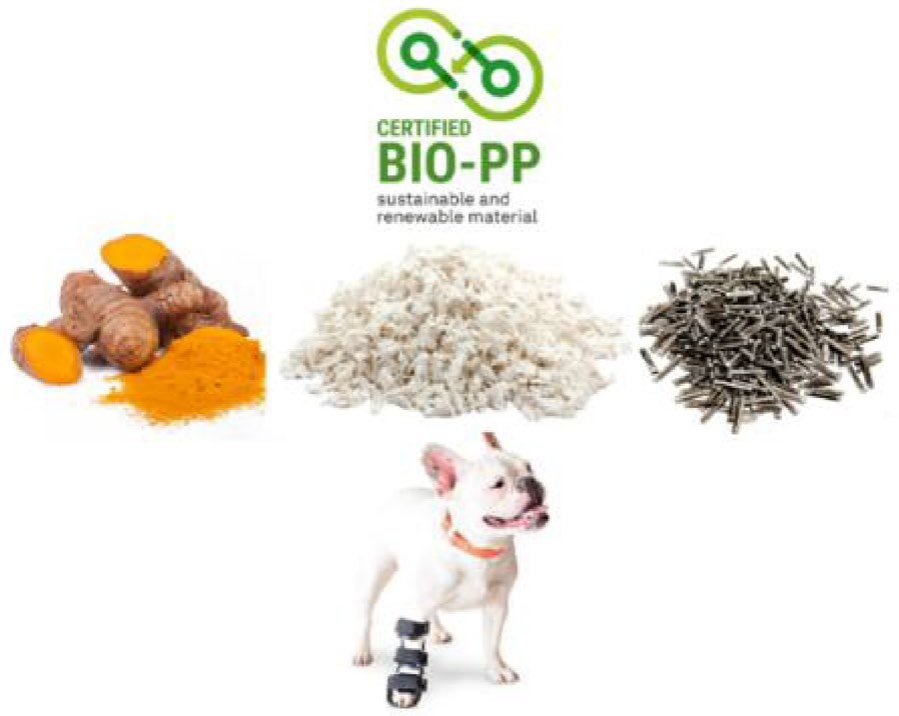
Production of small orthoses made of biobased PP with turmeric, lignocellulose and basalt fibers.

Figure 1 shows the idea of using novel composites with natural ingredients to produce a small dog orthosis. The use of natural and antibacterial ingredients will reduce the risk of potential infection of the affected area and minimize the tendency to irritation occurring with the use of today's orthoses made of polypropylene-glass fiber composites.

## Materials and methods

### Materials and composites preparation

Biobased polypropylene (NP BioPP 202-48) by NaturePlast (Ifs, France) was used as a matrix to produce the composites. This polymer has the following properties: biobased 30% max; density 0.90 g/cm^3^, Young’s modulus 1250 MPa, impact strength 5 kJ/m^2^ and thermal resistance 90 °C. The following additives were added to the matrix to produce the composites:

- basalt fibers (KV02M) produced by Basaltex (Wevelgem, Belgium) with diameter 17 μm, length 6.4 mm, density 2.67 kg/dm^3^, Young’s modulus 93–110 GPa, tensile strength 4150–4800 MPa;
- microcellulose fibers (FILTRACEL EFC 950), J. RETTENMAIER & SÖHNE GMBH (Rosenberg, Germany), is a chemithermomechanical pulp powder, fiber length 50–150 µm, density 110–145 g/l;
- powdered turmeric HEERA (P&B Foods Limited, UK), India origin;
- compatibilizer PP SCONA TPPP 9112 GA (MAPP), anhydride maleic by BYK Additives & Instruments (Wesel, Germany), MFR (190 °C, 2.16 kg) > 70–120 g/10 min, drying loss (3 h, 110 °C) < 0.5 wt.-%, MAH content ≥ 1 wt.-%, a dosage of 0.5–2% wt.-% is recommended.

Before processing bioPP pellets were dried in molecular dryer at 90 °C for 4 h.

Standard samples were produced by injection molding (KM 40–125 Winner Krauss Maffei, Krauss Maffei, Munich, Germany) according to the PN-EN ISO:3167 standard. On the biopolypropylene matrix six composites were produced with contained 5–15% by weight basalt fibers, 5–15 wt% microcellulose fibers, 2 wt% dried turmeric powder and 2% compatibilizer. The parameters of the injection process: temperatures in the zones: 140, 185, 200, 205, 210 °C, injection pressure: 800 bar and were the same for all composites. The used abbreviations of the produced composites are presented in Table1.

**Table 1:** Abbreviations and density of the bioPP composites.

Samples	Matrix	Basalt fibers [wt%]	EFC fibers [wt%]	Turmeric [wt%]	Compatibilizer [wt%]	Density [g/cm^3^]
PP	BioPP 202–48	–	–	–	–	0.90
PP5B		5	–	2	2	0.97
PP5EFC		–	5	2	2	0.81
PP5B5EFC		5	5	2	2	0.95
PP10B10EFC		10	10	2	2	1.02
PP15B15EFC		15	15	2	2	1.04

### Testing methods

#### Scanning electron microscope (SEM)

In order to assess the microstructure of the composites after the tensile test, microphotography was performed using a scanning electron microscope SEM (JEOL JSM-IT200, Tokyo, Japan) in low-vacuum at 20 kV at 50 ×, 500 × and 1000 × magnification. Before placing the composites in the electron microscope chamber, they were covered with gold particles using a sputter coater (DII-29030SCTR, JEOL, Tokyo, Japan).

#### Mechanical testing

The static tensile test and initial mechanical hysteresis loops were conducted using Shimadzu AGS-X 10 kN (Kyoto, Japan) testing machine. Tensile test was carried out in accordance with PN-EN ISO 527 standard. The crosshead speed was 5 mm/min. The test used cyclically loaded deformation to a maximum of 900 N (960 N is max. force for neat PP) to 400 N minimum and changes in maximum displacement were observed. The speed of load and unload specimens was 100 mm/min, which is translated into a low frequency of cycles and allows visualization of viscosity phenomena.

The three-point bending test was performed on the MTS Criterion 43 (Minnesota, United States) universal testing machine with the MTS software TestSuites 1.0 in accordance with PN-EN ISO 178 standard. The crosshead speed was10 mm/min.

The impact test was carried out using the Charpy method on a Zwick/Roell MTS-SP testing machine (Ulm, Germany), in accordance with the PN-EN ISO 179 standard. The samples were unnotched. The impact energy was 2 and 5J.

#### Water absorption

To determine the water absorbency of materials, the samples were placed in a saline solution at 40 °C for 6 weeks. Mass measurements were made after 24 h, and then after 7, 14, 21,28, 35 and 42 days in accordance with the PN-EN ISO 62:2008 standard. After drying, the samples were weighed using an Ohaus Adventurer laboratory balance (Parsippany, New Jersey, United States). Absorption was calculated according by following equation:

1 $${M}_{t}=\left[\frac{{W}_{t}-{W}_{0 }}{{W}_{0 }}\right]\times 100$$

where M_t_—percentage of water content, W_t_—instantaneous weight of the sample. W_0_—the initial weight of the sample, To assess the impact of degradation on the strength properties of the materials, the samples were subjected to tensile tests again after 6 weeks.

## Results and discussion

### Scanning electron microscope (SEM) micrographs

**Figure 2:**
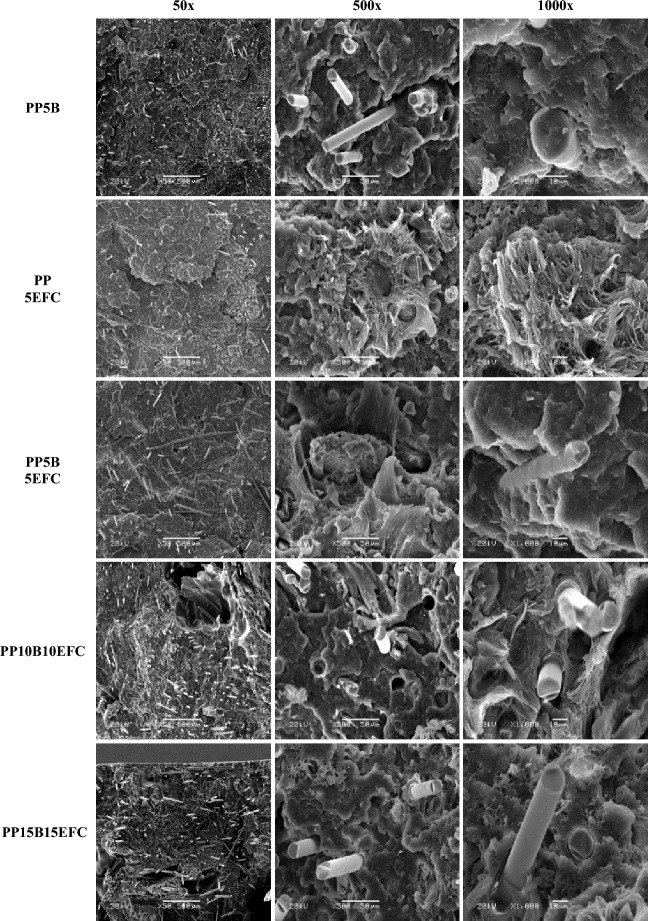
Comparison of SEM micrographs of the sample surface after tensile test of bioPP composites at 50 ×, 500 × and 1000 × magnification.

Figure 2 shows SEM micrographs of the samples surface after the tensile test for the 50 ×, 500 × and 1000 × of bioPP composites with basalt, EFC fibers and turmeric powder addition.

On the SEM micrographs of composites with the addition of 5% basalt fibers, we can observe their geometric dimensions, i.e., diameters ranging from 13 to 17 μm. Comparing all the fractures, we can estimate their length at about 1–1.5 mm. Basalt fibers are firmly embedded in the polypropylene matrix, the pull out phenomenon is almost not noticeable. The areas of crystalline clusters in micrographs with the addition of 5% basalt fibers are more characteristic at a size of approximately 50 μm, indicating the ability of the polypropylene matrix to crystallize around the fibers. The fibers are mostly aligned perpendicular to the tensile direction, indicating their partial orientation during the injection molding process and are arranged largely parallel to the direction of the injection axis. The small amount of voids formed when the fibers are pulled out indicates good adhesion of basalt fibers to polypropylene, which results in the noticeable increase in strength observed in the mechanical tests. In fractures after tensile test of composites with the addition of 5% EFC fibers we observe fine clusters of lignocellulosic fibrils and more developed surface of fractures in the case the addition of basalt. This results in an increase in deformation with a slight decrease in strength. Hybrid composites with equilibrium proportions of fibers 5/5, 10/10 and 15/15 are characterized by a different degree of mixing of cellulose and basalt fibers. The most homogeneous are those with a share of 10% of each filler.

### Mechanical properties

#### Static tensile and three-point bending tests

The results of mechanical properties from the static tensile and three-point bending tests are presented in Table 2.

**Table 2:** Results of tensile and bending tests.

Samples	Tensile test			Bending test		
	Tensile strength [MPa]	Young’s modulus [MPa]	Strain at break [%]	Bending strength [MPa]	Flexural modulus [MPa]	Strain at break [%]
PP	24.0 ± 0.3	1763 ± 151	26.60 ±0.25	34.6 ± 0.2	1214 ± 43	6.03 ± 0.32
PP5B	32.8 ± 1.7	2930 ± 170	4.36 ± 0.11	46.8 ± 2.1	1777 ± 210	5.95 ± 0.41
PP5EFC	28.3 ± 1.4	2284 ± 104	4.76 ± 0.02	43.9 ± 0.1	1692 ± 124	5.85 ± 0.23
PP5B5EFC	26.9 ± 1.2	2720 ± 89	2.99 ± 0.12	40.2 ± 1.2	2012 ± 175	5.99 ± 0.27
PP10B10EFC	35.2 ± 1.3	4437 ± 260	2.41 ± 0.05	49.8 ± 1.7	2689 ± 220	4.00 ± 0.16
PP15B15EFC	29.4 ± 1.6	4692 ± 248	1.55 ± 0.07	54.2 ± 2.2	3599 ± 301	2.76 ± 0.18

The addition of fibers increases tensile and flexural strength, modulus of elasticity and significantly reduces strain at break, especially the addition of basalt fibers.

The tensile strength of the composites with the addition of fibers was within the range of 26.9–35.2 MPa. The PP10B10EFC composite was characterized by the highest tensile strength. This value was 35.2 MPa, which is 32% higher than the lowest value (24 MPa) recorded for neat PP. The next highest value of tensile strength was obtained for PP5B and it was 32.8 MPa. The tensile strength of 15/15 composites increased by 22.5% and the Young's modulus by 166% compared to neat PP. Sergi et al. with the same fiber content and the same type of compatibilizer (maleic anhydride), achieved an improvement in these values by 45% and 284%, respectively^28^.

The addition of fiber significantly reduces strain at break value by 82–94%. The higher the fiber content in the composite, the lower the deformation at break.

The value of the flexural strength of the composites with the addition of fibers was within the range of 40.2–54.2 MPa. The addition of fibers increases the flexural modulus by 39–196% and is higher the higher the fiber content. A similar relationship as in the case of the tensile test can be seen in the bending test values. The flexural strength and modulus increased by 56.5% and 195.6%, where in the work of Sergi et al. these values changed by 97% and 263%, respectively, compared to neat PP. The higher percentage increase obtained by other authors is most likely due to the lack of the addition of turmeric, which reduces the strength properties^38^, and the higher addition of a compatibilizer (5%).

#### Mechanical hysteresis loops and energy dissipation effects

**Figure 3:**
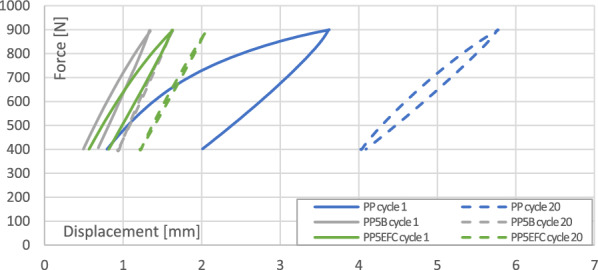
Comparison of the mechanical hysteresis loops for the first and the twentieth cycle for PP, PP with 5% basalt fibers and PP with 5% EFC fibers.

**Figure 4:**
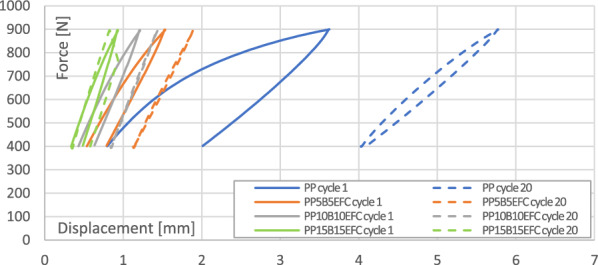
Comparison of the mechanical hysteresis loops for the first and the twentieth cycle for PP and its hybrid composites.

**Figure 5:**
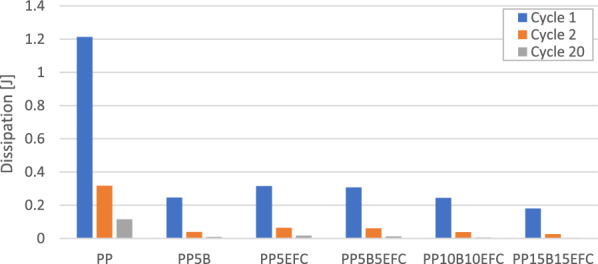
Comparison of ability to dissipate mechanical energy for the first, second and twentieth cycle of PP and its composites.

**Figure 6:**
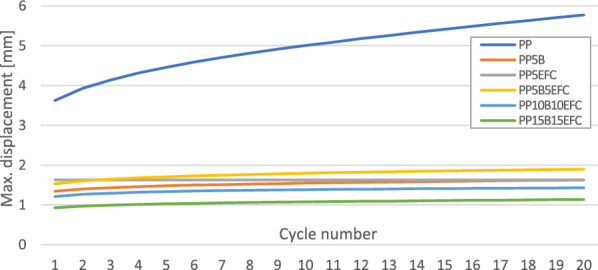
Comparison of the maximum displacement of PP composites on increasing loops of mechanical hysteresis.

Figures 3 and 4 present the mechanical hysteresis loops of biopolypropylene and its composites. Figure 5 shows the change in mechanical energy dissipation for the first, second and twentieth cycle and Fig. 6 the maximum displacement in the loop.

In polymer composites, decohesion processes (mainly at the interface) appear already during the first load cycles due to the fact that the polymer matrix is viscoelastic and the basalt fiber is elastic. During the deformation of the sample, stresses and strains do not occur in phase. We observe a certain delay of strain in relation to the applied stress, and consequently the appearance of a mechanical hysteresis loop. Dissipated energy is the difference between the energy converted mainly to heat and elastic potential energy during loading and the energy recovered during unloading.

We can observe that in subsequent cycles, the mechanical hysteresis loops decrease their field, i.e., the dissipation energy, which is related to the exhaustion of the ability to dissipate mechanical energy in subsequent cycles and in the next stages leads to fatigue failure. EFC more than basalt fibers increase the ability to dissipate mechanical energy as a result of better adhesion of the polymer matrix to the filler.

The maximum displacement in the loop indicates the intensity of relaxation processes, especially dynamic creep, and the likelihood of decohesion inside the material. The addition of basalt and EFC fibers significantly reduces the ability to creep and stabilizes the increase in maximum displacement after several cycles.

### Hydrothermal degradation

#### Water absorption

**Figure 7:**
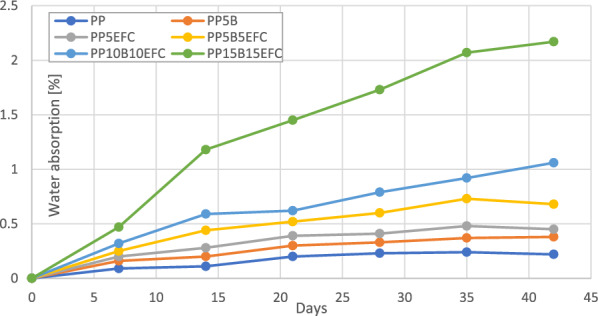
Water absorption during hydrothermal degradation of bioPP and its composites over a period of 6 weeks.

Figure 7 shows the water absorption of bioPP and its composites during hydrothermal degradation in a saline solution.

The largest mass gain was observed for the PP15B15EFC sample and after 42 days of hydrothermal degradation this value was 2.17%, which was almost 10 times higher compared to the lowest value for neat PP (0.22%). We can observe that EFC fibers increased water absorption more than basalt fibers. For each of the samples, the greatest increase in weight was observed in the first 14 days.

#### Influence of hydrothermal degradation on mechanical properties during tensile test

**Figure 8:**
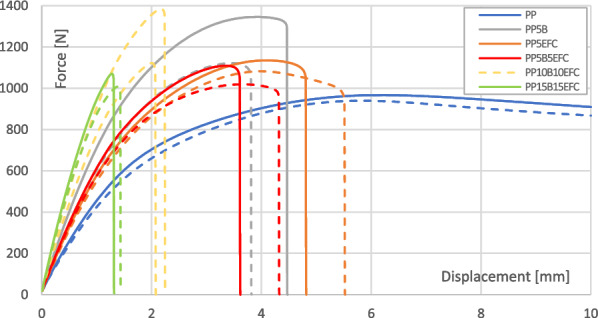
Static tensile test curves for PP composites before and after (dotted line) hydrothermal degradation.

**Figure 9:**
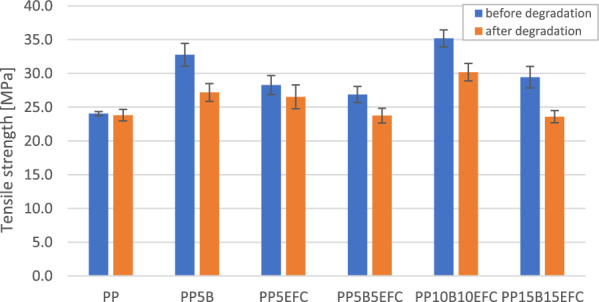
Comparison of tensile strength of PP composites before and after 6 weeks of hydrothermal degradation.

**Figure 10:**
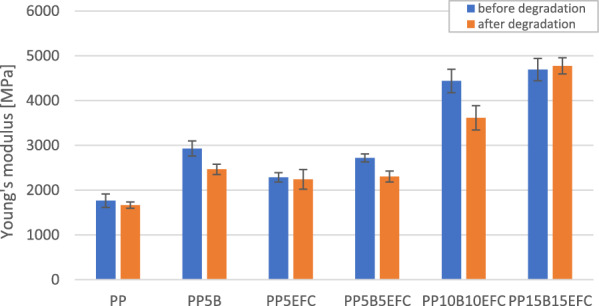
Comparison of Young’s modulus of PP composites before and after 6 weeks of hydrothermal degradation.

Figure 8 presents a comparison of curves from a static tensile test of PP and its composites before and after 6 weeks of hydrothermal degradation. Figures 9 and 10 show the influence of degradation on the change in tensile strength and Young’s modulus.

For each of the composites, we observe a decrease in tensile strength and Young’s modulus. The lowest value changes can be observed for neat PP. A greater decrease in the values occurs for composites with the addition of basalt fiber than for those with EFC fibers, probably as a result of increasing of volume of EFC which causes the water absorption along the fibers to stop.

#### Influence of hydrothermal degradation on mechanical properties during three-point bending test

**Figure 11:**
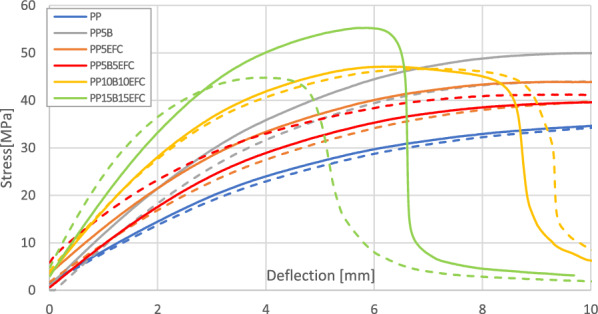
Three-point bending test curves for PP composites before and after (dotted line) hydrothermal degradation.

**Figure 12:**
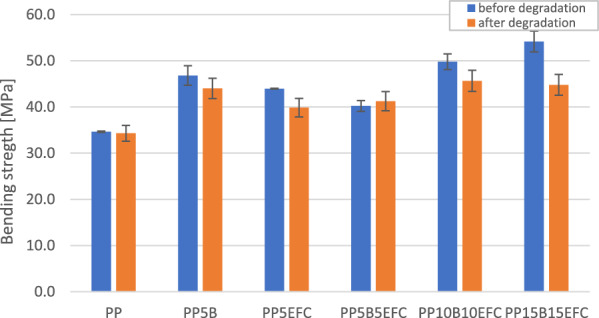
Comparison of bending strength of PP composites before and after 6 weeks of hydrothermal degradation.

**Figure 13:**
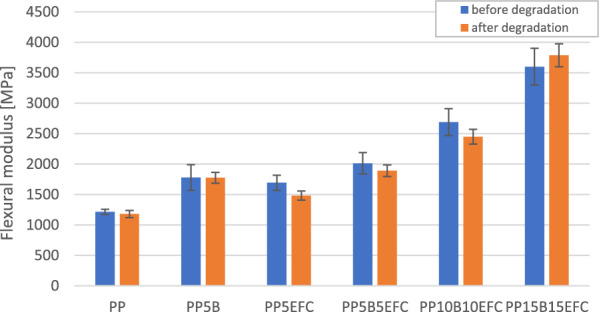
Comparison of flexural modulus of PP composites before and after 6 weeks of hydrothermal degradation.

A comparison of the curves from a three-point bending test of PP composites before and after 6 weeks of hydrothermal degradation is shown in Fig. 11. The influence of degradation on the change in bending strength and flexural modulus is presented on Figs. 12 and 13.

The characteristics of the bending curves change with the increasing amount of hybrid fillers. The greatest changes in the decrease in strength and the increase in flexural modulus are observed for composites with a share of 30 and 20% of the equilibrium amount of fibers.

The flexural strength of neat polypropylene did not change after hydrothermal degradation. For composites with the addition of fibers, this value slightly decreased, with the exception of the PP5B5EFC composite, for which the increase was within the limits of the measurement error. The greatest decrease in flexural strength was recorded for the PP15B15EFC composite, this value decreased by 17.3%. It can be concluded that the hydrothermal degradation of cellulose fibers and the polymer matrix increases with the increase in the amount of fibers.

The flexural modulus of biopolypropylene and composites changed slightly after 6 weeks of hydrothermal degradation. The addition of 5% EFC fibers reduced the modulus value by 12.4%. Similar changes were observed for composites with 5 and 10% basalt fibers. For the PP15B15EFC composites, the flexural modulus increased by about 5.2%, probably as a result of the large amount of fillers in the composition.

#### Influence of hydrothermal degradation on mechanical properties during impact test

**Figure 14:**
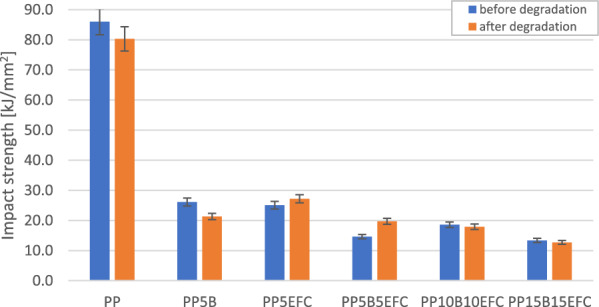
Comparison of impact strength of PP composites before and after 6 weeks of hydrothermal degradation.

Figure 14 shows changes in the impact strength of injected composites before and after the degradation process depending on the proportions of fillers.

The impact strength of neat polypropylene, after 6 weeks of hydrothermal degradation, slowly decreases, probably as a result of leaching of the remains of the bio-based matrix. Even a small 5% addition of EFC causes an increase in impact strength, probably due to the increase in the volume of cellulose fibers under the influence of water sorption and stress between the fiber and the polymer matrix. The addition of basalt fibers increases the water sorption at the interface between the fiber and the polymer.

## Conclusion

The presented research results confirm that it was possible to produce novel functional biocomposites based on polypropylene from biomass with the addition of basalt and lignocellulosic fibers modified with natural antibacterial turmeric. The test results indicate the possibility of improving the strength both by the addition of basalt fibers and the stiffness by cellulose microfibers. The higher the filler content in the form of basalt fibers and cellulose fibers in the composite, the higher the bending strength. Basalt fibers increase flexural strength more than lignocellulosic fibers as their amount in the composite increases. The most promising seem to be hybrid composites with a balanced proportion of 10:10 and 15:15 basalt and EFC fibers, which are characterized by 20% higher strength and almost two and a half times higher stiffness than neat polypropylene. Stiffness in many applications, such as small orthoses, supports, is essential for the healing process.

Summarizing the results of the study of strength properties, it can be noted that the addition of basalt fibers increases strength and stiffness, and microcellulose particles reduce the ability to dissipate mechanical energy, while in either case water has a plasticizing effect on the produced composites. In addition, such composites, compared to those reinforced with glass fibers, have smooth surfaces and unlike glass fibers with polypropylene composites, do not cause irritation and abrasions. This is especially important when treating children and small animals whose skin is thin and delicate. Such composites are also characterized by a lower density than those with only glass fiber, thanks to which the manufactured elements of medical equipment will be lighter than traditional ones. In further studies, it is proposed to make a prototype by injection molding of the rabbit orthosis and evaluate the histopathological compatibility of the composite and the tendency to irritation in comparison with a traditional orthosis made of polypropylene with glass fiber. In parallel, long-term fatigue properties and thermal tests of the rendered composites can be carried out to optimize processing conditions and learn about the effect of natural additives on changes in the crystallinity of the composite.

The datasets used and/or analysed during the current study available from the corresponding author on request.
